# Trends in and Predictions of Colorectal Cancer Incidence and Mortality in China From 1990 to 2025

**DOI:** 10.3389/fonc.2019.00098

**Published:** 2019-02-21

**Authors:** Lei Zhang, Fei Cao, Guoyao Zhang, Lei Shi, Suhua Chen, Zhihui Zhang, Weiguo Zhi, Tianjiang Ma

**Affiliations:** Department of Oncology, Luohe Central Hospital, Luohe, China

**Keywords:** colorectal cancer, incidence, mortality, prediction, China

## Abstract

Colorectal cancer (CRC) has emerged as a major public health concern in China during the last decade. In this study, we investigated the disease burden posed by CRC and analyzed temporal trends in CRC incidence and mortality rates in this country. We collected CRC incidence data from the Cancer Incidence in Five Continents, Volume XI dataset and the age-standardized incidence rate (ASIR) and age-standardized mortality rate (ASMR) of CRC by sex and age, from the 2016 Global Burden of Diseases Study. We used the average annual percentage change (AAPC) to quantify temporal trends in CRC incidence and mortality from 1990 to 2016 and found the ASIR of CRC increased from 14.25 per 100,000 in 1990 to 25.27 per 100,000 in 2016 (AAPC = 2.34, 95% confidence interval [CI] 2.29, 2.39). Cancer cases increased from 104.3 thousand to 392.8 thousand during the same period. The ASIR increased by 2.76% (95% CI 2.66%, 2.85%) and 1.70% (95% CI 1.64%, 1.76%) per year in males and females, respectively. The highest AAPC was found in people aged 15–49 years (2.76, 95% CI 2.59, 2.94). Cancer deaths increased from 81.1 thousand in 1990 to 167.1 thousand in 2016, while the ASMR remained stable (−0.04, 95% CI −0.13, 0.05), A mild increase (AAPC = 0.42, 95% CI 0.34, 0.51) was found among males and a significant decrease (AAPC = −0.75, 95% CI −0.90, −0.60) was found among females. Between 2016 and 2025, cancer cases and deaths are expected to increase from 392.8 and 167.1 thousand in 2016 to 642.3 (95% CI 498.4, 732.1) and 221.1 thousand (95% CI 122.5, 314.8) in 2025, respectively. Our study showed a steady increase in the CRC incidence in China over the past three decades and predicted a further increase in the near future. To combat this health concern, the prevention and management of known risk factors should be promoted through national polices. Greater priority should be given to CRC prevention in younger adults, and CRC screening should be widely adopted for this population.

## Introduction

Colorectal cancer (CRC) is a common diagnosed malignant neoplasm, which ranks third among all cancers in terms of incidence and second in terms of mortality (1). In 2018, nearly 2.0 million newly diagnosed CRC cases and more than 0.8 million related deaths are expected to occur worldwide (1). CRC incidence rates vary substantially across the world, with the highest rates observed in parts of Europe (e.g., Slovakia, the Netherlands, Norway, and Hungary), in which the age-standardized incidence rates (ASIRs) have been as high as 60 per 100,000 for males and 35 per 100,000 for females (2), but CRC ASIRs in Asia have been two to three-fold lower than that in Europe (2, 3). The striking disparity in the global incidence of CRC reflects the strong impact of lifestyle factors on the occurrence of this cancer (4). Moreover, variations in genetic background between different populations, such as single nucleotide polymorphisms in certain genes, have been hypothesized to be associated with CRC genesis (5–7). CRC incidence and mortality rates have been stabilizing or decreasing in highly developed countries through the enormous efforts over the last decades (8). However, a rapid upward trend has been seen in many low-income and middle-income countries (4).

China has been the world's fastest-growing developing country for the past four decades. Urbanization, aging population, and shift to sedentary lifestyle and Westernized diet has led to a shift in the disease burden from infectious to non-communicable diseases (9). One of the major public health issues is the rapid rise in CRC incidence and the accompanying increase in disease burden (10, 11). According to cancer statistics for 2010 (12), ~274.8 thousand new CRC cases and 132.1 thousand CRC related deaths were estimated to occur in China, accounting for nearly one tenth of the global CRC burden. Knowing the epidemiological features of CRC, including its temporal trends and geographical patterns in China, therefore, is critical for both the prevention and management of cancer in this country.

This study used the CRC incidence and mortality data of China from the Global Burden of Diseases (GBD) 2016 Study and the data from the Cancer Incidence in Five Continents Volume XI of the International Agency for Research on Cancer. Both databases provide us with a unique opportunity to understand the CRC landscape in China. We described the contemporary geographical distribution of CRC incidence and assessed the temporal trends in its incidence and mortality from 1990 to 2016. Moreover, we conducted a forecasted CRC incidence and mortality up to 2025. The results yielded by our study should be useful for the assessment of current prevention strategies, to enhance our understanding of and planning to manage the disease burden, and to promote the evolution of China's Health System to respond to future challenges.

## Materials and Methods

### Study Data

**Figure 1:**
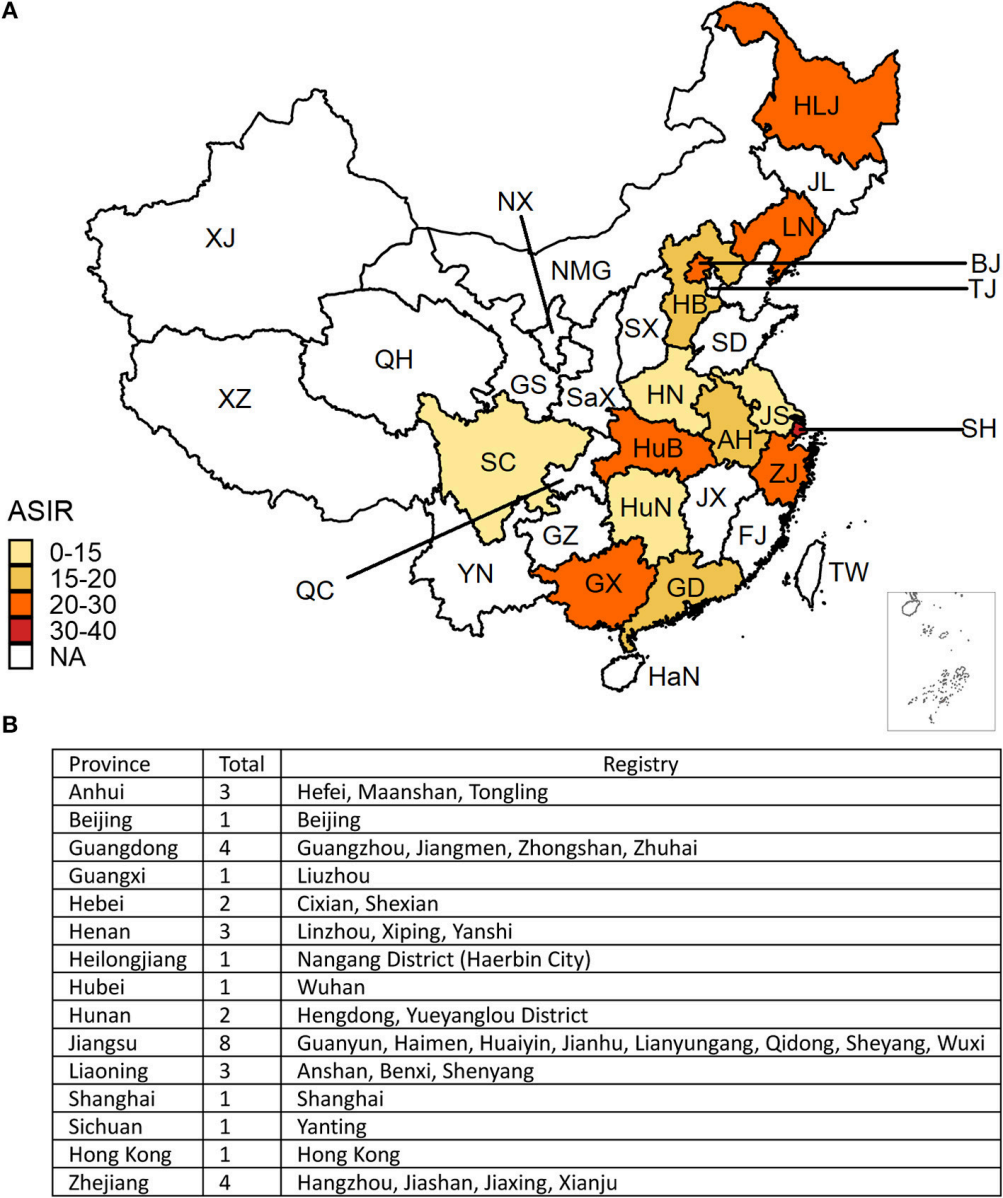
**(A)** The geographical distribution of the age-standardized incidence rate (ASIR) per 100,000 colorectal cancer cases in China. The deeper the color, the higher the rate. The white areas indicate no data were mapped. **(B)** The cancer registries included in this analysis. (BJ, Beijing; TJ, Tianjin; HB, Hebei; NMG, Inner Mongolia; SX, Shanxi; HLJ, Heilongjiang; JL, Jilin; LN, Liaoning; SD, Shandong; JS, Jiangsu; SH, Shanghai; ZJ, Zhejiang; AH, Anhui; FJ, Fujian; JX, Jiangxi; GD, Guangdong; GX, Guangxi; HaN, Hainan; YN, Yunnan; GZ, Guizhou; XZ, Tibet; SC, Sichuan; QC, Chongqing; HuN, Hunan; HuB, Hubei; HN, Henan; SaX, Shaanxi; GS, Gansu; NX, Ningxia; QH, Qinghai; XJ, Xinjiang).

We collected CRC incidence data from the Cancer Incidence in Five Continents Volume XI (CI5XI) dataset (13), which contains the average annual incidence of cancer (2008–2012) by sex, site, and age-group per 100,000 population from 36 cancer registries in China (Figure 1B, the table embedded in the Figure 1). The codes from 10th revision of the International Statistical Classification of Diseases and Related Health Problems (2010) corresponding to CRC (colon cancer: C18; rectal cancer: C19-20) were used to identify cancer cases. We combined colon cancer and rectal cancer to estimate the overall ASIR of CRC in different regions in China. We merged the data and provided a summary estimate for the provinces with several cancer registries. The ASIRs of CRC were plotted on a national map. We retrieved annual incident cases, ASIRs, mortality, age-standardized mortality rates (ASMRs), and Disability-Adjusted Life Years (DALYs), Years Lived with Disability (YLDs), and Years with Life Lost (YLLs) of CRC from 1990 to 2016, by sex and age group from the Global Health Data Exchange, a comprehensive online catalog of the GBD data (http://ghdx.healthdata.org/gbd-results-tool) (14). The cancer incidence was sought from individual cancer registries or aggregated databases of cancer registries, including Cancer Incidence in Five Continents (CI5) (15) and SEER (Surveillance, Epidemiology, and End Results) (16). Like data in CI5XI database, all cancer data stored in GBD were stratified by cancer site, country or region, age group, sex, and time. The general methods used in the 2016 GBD Study and the methods for estimating the disease burden of CRC have been detailed in previous studies (17). We matched all neoplasms as defined in the International Statistical Classification of Diseases and Related Health Problems to one of the 29 GBD cancer groups (18, 19). In the GBD Study, 95% uncertainty intervals (95% UI) were calculated to provide information on the variability of estimates resulting from errors related to the sampling process, and to non-sampling errors related to adjustments to data sources and modeling (18).

### Statistical Analysis

The Standard World Population 2000 was used to estimate ASIRs of CRC per 100,000 person years for all regions. We used the average annual percentage change (AAPC) to quantify temporal trends in CRC incidence and mortality from 1990 to 2016 (20). A regression line was fitted to the natural logarithm of the rates, i.e., *y* = α + β*x* + ε, where *y* = ln(ASIR or ASMR), and *x* = calendar year. The AAPC was calculated as 100 × (*exp*(β)-1). The 95% confidence interval (CI) of the AAPC was also calculated in the regression model. The ASIR (ASMR) showed an upward trend when the AAPC estimation and the lower boundary of its 95% CI were both > 0. In contrast, the ASIR (ASMR) showed a downward trend when the AAPC estimation and the upper boundary of its 95% CI were both < 0. Otherwise, the ASIR (ASMR) was deemed to be stable over time (21). For a clearer depiction of the temporal trend of the ASIR, we divided the study period into two discrete timeframes (1990–2010 and 2011–2016) and assessed the ASIR trend for each timeframe. The study period for the ASMR was also separated into two discrete periods (1990–2002 and 2003–2016), and the AAPC for each timeframe was also assessed separately. Furthermore, we predicted the numbers of cases and deaths from CRC between 2017 and 2025 by conducting a Bayesian age-period-cohort analysis using integrated nested Laplace approximations, which has been well-documented and validated in other studies (22, 23). All statistical analyses were performed using the R program (Version 3.4.1, R core team). The Bayesian age-period-cohort model was analyzed using the BAPC (Bayesian age-period-cohort) and INLA (integrated nested Laplace approximation) packages in R. A *P* < 0.05 was considered statistically significant.

## Results

**Figure 2:**
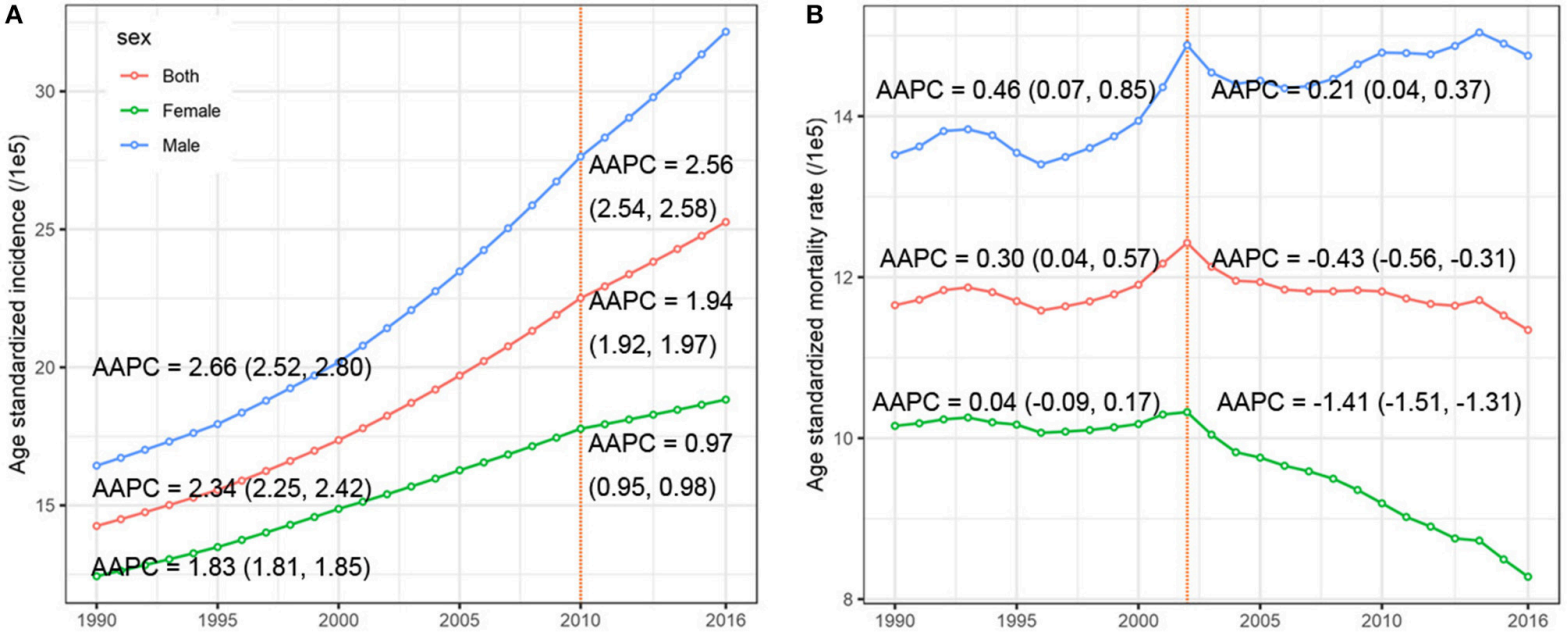
The temporal trends of the **(A)** age-standardized incidence rate, **(B)** age-standardized mortality rate, by sex, from 1990 to 2016. The AAPCs presented in **(A,B)** were derived from a period-specific data: **(A)** 1990–2010 and 2011–2016; **(B)** 1990–2002 and 2003–2016.

The CRC incidence data retrieved from the 36 cancer registries in 15 provinces were included in the study (Figure 1). The combined CRC ASIRs from 2008 to 2012 showed significant variation across the country, with the highest ASIR observed in Hong Kong (39.97 per 100,000), followed by Shanghai (32.30 per 100,000), Liaoning (29.62 per 100,000), and Zhejiang (24.30 per 100,000). At the national level, the ASIR of CRC increased from 14.25 (95% UI 13.75, 14.93) per 100,000 in 1990 to 25.27 (95% UI 24.02, 26.47) per 100,000 in 2016, with an overall AAPC of 2.34 (95% CI 2.29, 2.39). Cancer cases increased nearly three-fold from 104.3 thousand (95% UI 100.6, 109.4) to 392.8 thousand (95% UI 373.0, 412.4) during the same period (Table 1). CRC cases and ASIRs were significantly higher in males than females. Among males, the ASIR increased by 2.76% (95% CI 2.66, 2.85) per year from 1990 to 2016, and the cancer cases increased more than three times compared to 1990. Among females, the ASIR of CRC increased from 12.43 per 100,000 to 18.83 per 100,000, with an AAPC of 1.70 (95% CI 1.64, 1.76). Cancer cases among females increased from 46.9 thousand to 148.9 thousand during the study period (Table 1). The specific timeframes of the AAPCs in the ASIRs are presented in Figure 2A. In general, the CRC ASIRs of both males and females rapidly increased from 1990 to 2010; thereafter, the rising trends slowed. We also examined the CRC ASIRs among the three age groups. The highest ASIR was observed among individuals aged 50–69 years in terms of number of cancer cases and in those aged 70+ years in terms of the ASIR. However, the highest AAPC was found in people aged 15–49 years (2.76, 95% CI 2.59, 2.94) (Table 1).

**Table 1:** The cancer cases and age standardized incidence rate of colorectal cancer in China in 1990 and 2016.

	**1990**		**2016**		**1990–2016**	
	**Cancer cases**	**ASIR (/1e5)**	**Cancer cases**	**ASIR (/1e5)**	**Change in cancer cases (%)**	**AAPC in ASIR**
	**No. × 10^**3**^ (95% UI)**	**No. × 10^**3**^ (95% UI)**	**No. × 10^**3**^ (95% UI)**	**No. × 10^**3**^ (95% UI)**		**No. (95% CI)**
Overall	104.3 (100.6, 109.4)	14.25 (13.75, 14.93)	392.8 (373.0, 412.4)	25.27 (24.02, 26.47)	276.6 (257.4, 289.3)	2.34 (2.29, 2.39)
**SEX**						
Male	57.5 (55.3, 59.7)	16.44 (15.81, 17.07)	243.9 (234.4, 253.1)	32.16 (31.00, 33.33)	324.2 (300.8, 350.1)	2.76 (2.66, 2.85)
Female	46.9 (44.4, 50.7)	12.43 (11.78, 13.42)	148.9 (135.8, 162.7)	18.83 (17.16, 20.52)	217.5 (203.2, 234.3)	1.70 (1.64, 1.76)
**AGE (YEARS)**						
5–14	0	0	0	0	–	–
15–49	21.6 (20.6, 22.7)	3.35 (3.19, 3.53)	54.6 (51.0, 59.3)	7.32 (6.84, 7.95)	152.3 (129.4, 171.4)	2.76 (2.59, 2.94)
50–69	49.5 (47.5, 52.2)	34.33 (32.98, 36.18)	201.2 (190.7, 212.5)	62.71 (59.42, 66.22)	306.5 (278.6, 332.4)	2.34 (2.21, 2.47)
70+	33.2 (31.9, 34.9)	87.84 (84.30, 92.32)	137.0 (130.2, 143.3)	163.3 (155.3, 170.9)	312.7 (297.4, 340.8)	2.57 (2.50, 2.64)

*UI, uncertainty interval; ASIR, age standardized incidence rate; AAPC, average annual percentage change; CI, confidence interval*.

**Figure 3:**
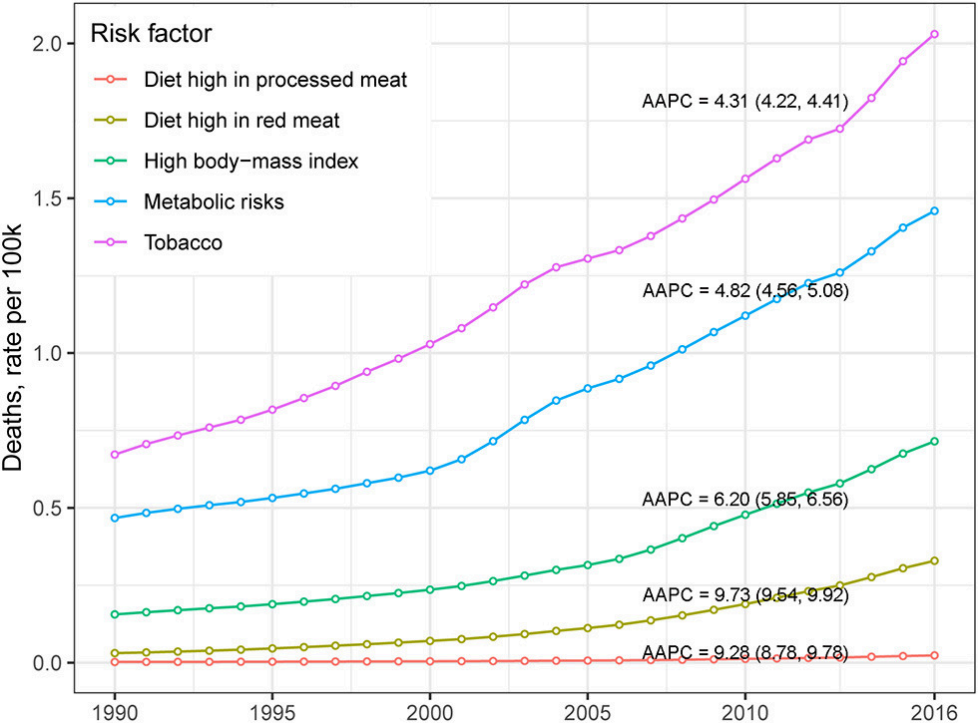
The temporal trends of CRC death rate, stratified by risk factor, in China from 1990 to 2016.

Table 2 presents the ASMR and cancer deaths from CRC in 1990 and 2016. Overall, cancer deaths increased from 81.1 thousand in 1990 to 167.1 thousand in 2016, whereas the ASMR remained stable (AAPC = −0.04, 95% CI −0.13, 0.05) during the same period. For males, a mild increase (AAPC = 0.42, 95% CI 0.34, 0.51) was observed in the ASMR during 1990-2016. In contrast, a significant decrease (AAPC = −0.75, 95% CI −0.90, −0.60) was found in females. The AAPC in the CRC ASMR was time dependent. As shown in Figure 2B, an increase in the overall ASMR was observed from 1990 to 2002, whereas the ASMR decreased thereafter (AAPC = −0.43, 95% CI −0.56, −0.31). The ASMR increased among males by 0.46 per year from 1990 to 2002, and the rising trend slowed from 2003 to 2016. Among females, a stable trend in the ASMR was found between 1990 and 2002. However, the ASMR showed a downward trend after 2002. The significant increase was detected only among people aged 70+ years (AAPC = 0.51, 95% CI 0.38, 0.63) (Table 2). We also retrieved the data on risk factors (including tobacco use, metabolic risks, high body-mass index [BMI], diet high in red meat, and diet high in processed meat) for CRC mortality during the study period. The temporal trends in the risk factors contribution for CRC mortality were estimated (Figure 3). Among the five well-established risk factors, the most significant contribution was tobacco use, followed by metabolic risks, high BMI, diet high in red meat, and diet high in processed meat. In contrast, the most pronounced increases in CRC death rate was found in diet high in red meat and diet high in processed meat.

**Table 2:** The deaths and age standardized mortality rate of colorectal cancer in China in 1990 and 2016.

	**1990**		**2016**		**1990-2016**	
	**Deaths**	**ASMR (/1e5)**	**Deaths**	**ASMR (/1e5)**	**Change in deaths (%)**	**AAPC in ASMR**
	**No. × 10^**3**^ (95% UI)**	**No. × 10^**3**^ (95% UI)**	**No. × 10^**3**^ (95% UI)**	**No. × 10^**3**^ (95% UI)**	**No. (95% CI)**	**No. (95% CI)**
Overall	81.1 (77.2, 85.7)	11.65 (11.10, 12.28)	167.1 (159.1, 174.5)	11.34 (10.81, 11.83)	106.0 (97.8, 111.6)	−0.04 (-0.13, 0.05)
**SEX**						
Male	44.4 (42.2, 46.7)	13.52 (12.89, 14.16)	104.2 (99.6, 109.0)	14.75 (14.11, 15.38)	134.7 (126.5, 140.7)	0.42 (0.34, 0.51)
Female	36.7 (34.1, 40.1)	10.15 (9.42, 11.09)	62.9 (57.6, 68.2)	8.28 (7.60, 8.98)	71.4 (63.2, 79.6)	−0.75 (−0.90, −0.60)
**AGE (YEARS)**						
5–14	0	0	0	0	–	–
15–49	14.5 (13.8, 15.3)	2.25 (2.14, 1.38)	17.6 (16.5, 18.7)	2.36 (2.21, 2.51)	21.4 (14.6, 26.5)	−0.31 (−0.55, −0.07)
50–69	36.3 (34.6, 38.5)	25.20 (23.96, 26.74)	73.7 (70.0, 77.4)	22.98 (21.83, 24.12)	103.0 (90.3, 112.3)	−0.37 (−0.46, −0.27)
70+	30.2 (28.7, 32.2)	80.00 (75.99, 85.08)	75.7 (72.2, 79.0)	90.32 (86.05, 94.14)	150.7 (145.3,156.1)	0.51 (0.38, 0.63)

*UI, uncertainty interval; ASMR, age standardized mortality rate; AAPC, average annual percentage change; CI, confidence interval*.

**Figure 4:**
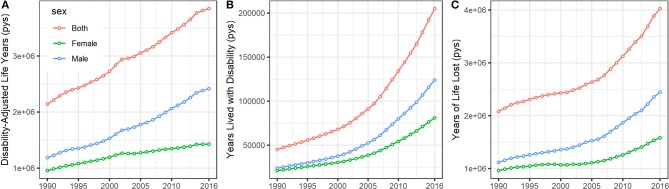
The temporal trends of the **(A)** Disability-Adjusted Life Years (DALYs), **(B)** Years Lived with Disability (YLDs), and **(C)** Years with Life Lost (YLLs) of CRC stratified by sex in China from 1990 to 2016. (pys, person years).

The annual Disability-Adjusted Life Years (DALYs), Years Lived with Disability (YLDs), and Years with Life Lost (YLLs) of CRC stratified by sex are presented in Figure 4. Overall, the disease burden posed by CRC was substantially increased in China during the study period. Specifically, the DALYs, YLDs, and YLLs increased 79.4, 354.4, and 64.5, respectively, between 1990 and 2016.

**Figure 5:**
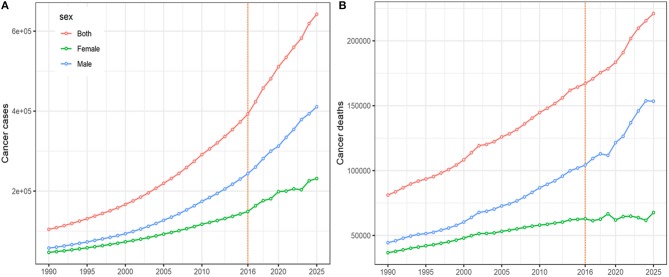
The projections of colorectal cancer cases **(A)** and deaths **(B)**, by sex, from 2017 to 2025 in China.

The results of the forecast of CRC cases and deaths for 2017–2025 based on the data from the GBD Study are shown in Figure 5. New CRC cases and deaths will continue to rise in the near future. Cancer cases among males will increase from 243.9 thousand in 2016 to 410.9 thousand (95% CI 398.4, 422.4) in 2025, and the corresponding cancer-related deaths will increase from 104.2 thousand to 153.4 thousand (95% CI 142.2, 164.8) during this period. Among females, cancer cases will increase from 148.9 thousand in 2016 to 231.4 thousand (95% CI 213.2, 253.7) in 2025. Accordingly, the number of deaths caused by CRC will increase from 62.9 thousand to 67.7 thousand (95% CI 63.2, 73.7).

## Discussion

Colorectal cancer is one of the most common malignancies diagnosed worldwide (1). In this study, we analyzed the disease burden caused by CRC and temporal trends in CRC incidence and mortality in China. In general, the CRC incidence was relatively lower than that of developed countries (4, 24–26). However, this study showed an upward trend in its incidence, although there has been a downward trend in the mortality of CRC during the past three decades. The CRC incidence and mortality in males were almost twice as high as that of females. The most pronounced increase in the incidence of CRC was observed in younger and middle-aged adults.

Risk factors for CRC have been investigated extensively in the last decade. Previous epidemiological studies have suggested that alcohol consumption, diets high in meat intake, obesity, smoking, and physical inactivity are associated with an increased risk of CRC in various populations (27–31). Targeted prevention strategies for combating CRC have led to a significant decrease in its incidence in several developed countries (32, 33). However, an upward trend has been observed in most developing countries (4). Likewise, in this study, we found that CRC incidence has rapidly increased from 1990 to 2016 and will further increase in China over the next decade. This remarkable increase might be attributed mainly to the upward trend in Western dietary patterns (34), changes in occupational patterns (35), increases in high-risk behaviors (e.g., smoking and excessive calorie intake) (36), and an increase in the aging population (3). Moreover, changes in access to health care and early cancer screenings might contribute to variations in CRC incidence (37). In a report of a longitudinal analysis of dietary patterns of Chinese adults from 1991 to 2009, the authors expressed concern about the increasing popularity of the modern high-wheat dietary pattern, which consists of energy-dense foods, in the context of China's rapid economic changes (38). A Westernized diet, characterized by high intake in red meat and processed meat (39), might drive changes in cancer profiles, including an increase in CRC (34), which has been verified by epidemiological studies on immigrants (40, 41). For example, the CRC incidence among Asian migrants to the US has shifted to an intermediate level between their country of origin and their new country of residence (40, 41). The shift in diet pattern and the concurrently increase in CRC incidence rate in Chinese population emphasizes the importance of establishment of healthy diet for CRC prevention. In this regard, the general population might be benefitted more from a traditional Chinese diet or Mediterranean diet pattern (42, 43). Moreover, the prevalence of known risk factors, such as obesity, diabetes, and smoking, are still on the rise (44–47), although measures have been initiated to counter these risks. As a result, the incidence of CRC is expected to increase in the future as predicted in this study if effective interventions are not introduced.

Surprisingly, we found that the most pronounced increase in CRC incidence was observed in people aged 15–49 years. This finding is consistent with those of countries in which similar trends in younger populations have been reported over roughly the same period (48). For instance, in the US, the incidence rates of CRC among people below 50 years of age rose 1.61% per year in men and 1.46% per year in women from 1998 to 2009 (49). As more than 80% of young-onset CRC cases are diagnosed with symptomatic disease, which may be associated with a delay in diagnosis and poor prognosis (50), greater emphasis should be placed on young adults' risk reduction and awareness of CRC symptoms, and greater vigilance by healthcare providers is needed for the early detection of CRC in young patients.

Over the past two decades, the range of CRC screening modalities has expanded, and many population-based programs have been initiated (51). As expected, screening programs have been more frequently implemented in Western countries with higher rates of CRC and more available resources. Recommendations for CRC screening in the Asia Pacific region have been published and recently updated (52). Currently, those aged 40–74 years in China are screened using the fecal occult blood test, and followed up with a digital rectal exam and colonoscopy, which might influence the temporal trends in CRC, especially in the recent years. However, this program is far from complete and the national registry used to track clinical outcomes captures only 13% of the country's population, increasing the difficulty of planning healthcare services (53). The screening method also have impact on the identification of CRC subtypes. For example, more sigmoidoscopy screening would identify more rectal cases than colonoscopy does, though these screening methods have not widely adopted in China due to their cost and availability. In addition, screening methods with improved diagnostic ability or more sensitive biomarkers, such as methylated *Septin9* (m*SEPT9*), have been proposed and developed (54), though these potential biomarkers might be expensive and also not recommended by the current global guidelines or Asian-pacific guidelines for CRC screening.

Although the incidence of CRC is on the rise, we have observed a mild decrease in the CRC mortality rate, especially in recent years. This decrease might involve at least two aspects. First, the implementation of CRC screening to detect cancer in the earlier stages has demonstrated its effectiveness in populations that have initiated early screening programs. Second, the clinical treatment and management of CRC patients has received substantial improvement. For example, the 5-year survival rate has increased from 49% in the 1960s to 77% in the 2000s among CRC patients (55). The unequilibrium between CRC incidence rate and mortality rate can be partly explained by these reasons. Other factors contributed to this unequilibrium warrant further investigations. Of note, cancer-related deaths have doubled between 1990 and 2016 and are expected to increase in the near future, which might be the results of population expansion and aging. CRC, therefore, is a major public health concern, and poses a heavy disease burden on China.

Our study has limitations. First, we used multiple data sources that represented a diverse population. The data were retrieved from different international agencies; therefore, incompatibilities between these data might exist. Second, changes in risk factors and population structures were not taken into account when we predicted the cancer cases and deaths in future years. Finally, since the diagnostic pattern of CRC depends strongly on the screening compliance in the population and the screening method might also have effect on the disease form, the results presented in our study might be interpreted with cautions.

In summary, our study presents evidence of a steady increase in CRC incidence in China over the past three decades. It also predicts further increases in the near future. The sample's diversity of age, gender, and geography may yield insights for the future development of national programs. For example, the prevention and management of known risk factors, e.g., obesity and smoking, should be improved through effective national polices. Efforts should be made to prioritize CRC prevention in younger adults, as the incidence of CRC has increased in this group, and it could be prevented through the adoption of screening programs for this population.

## Author Contributions

TM: study design; LZ, FC, GZ, LS, SC, ZZ, and WZ: data collection; LZ and GZ: data analyses; all authors: results interpretations; LZ, FC, GZ, LS, SC, ZZ, and WZ: manuscript writing; TM and WZ: manuscript proofing.

### Conflict of Interest Statement

The authors declare that the research was conducted in the absence of any commercial or financial relationships that could be construed as a potential conflict of interest.

## Abbreviations

CRC: colorectal cancer
ASIR: age-standardized incidence rate
ASMR: age-standardized mortality rate
AAPC: average annual percentage change
GBD: global burden of diseases.
